# Multi-hole two-way hybrid placement using three 8-mm metal stents for malignant hilar biliary obstruction

**DOI:** 10.1055/a-2845-1766

**Published:** 2026-04-20

**Authors:** Hirotsugu Maruyama, Yoshinori Shimamoto, Yuji Kawata, Tatsuya Kurokawa, Yuki Ishikawa-Kakiya, Kojiro Tanoue, Yasuhiro Fujiwara

**Affiliations:** 1 12935 Department of Gastroenterology, Osaka Metropolitan University Graduate School of Medicine, Osaka, Japan

Multidisciplinary treatment for malignant hilar biliary obstruction (MHBO) has improved, and biliary drainage has become increasingly important. Liver drainage of more than 80% is associated with prolonged survival 1 ; however, the placement of three or more metal stents is technically challenging and therefore not commonly performed. Nevertheless, such extensive stenting may be required in cases of cholangitis or insufficient biliary drainage. Uncovered self-expandable metal stents (SEMSs) are generally used; however, they cannot be removed, and sequential stent insertion through the mesh becomes difficult. Herein, we report a case of MHBO with insufficient biliary drainage in which adequate drainage was successfully achieved by hybrid placement using three multi-hole self-expandable metal stents (MHSEMSs).

**Fig. 1:**
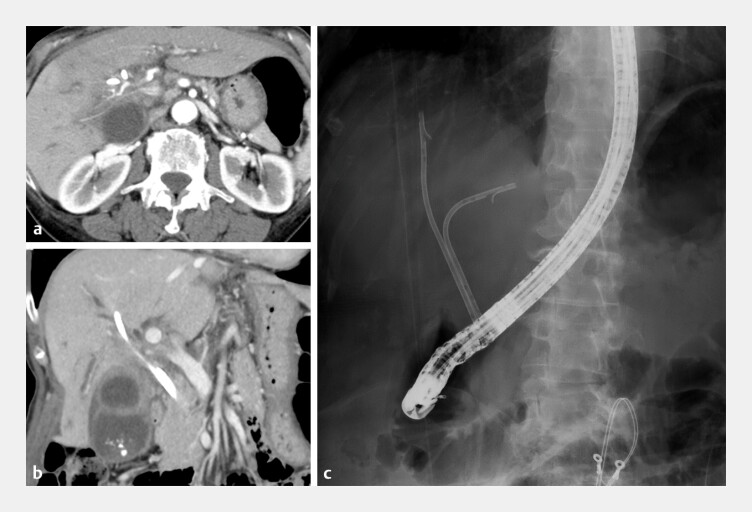
Computed tomographic (CT) and fluoroscopic images. **a** and **b** Axial and coronal images show hilar cholangiocarcinoma and an indwelling plastic stent. **c** A fluoroscopic image demonstrates two previously placed inside plastic stents.

**Fig. 2:**
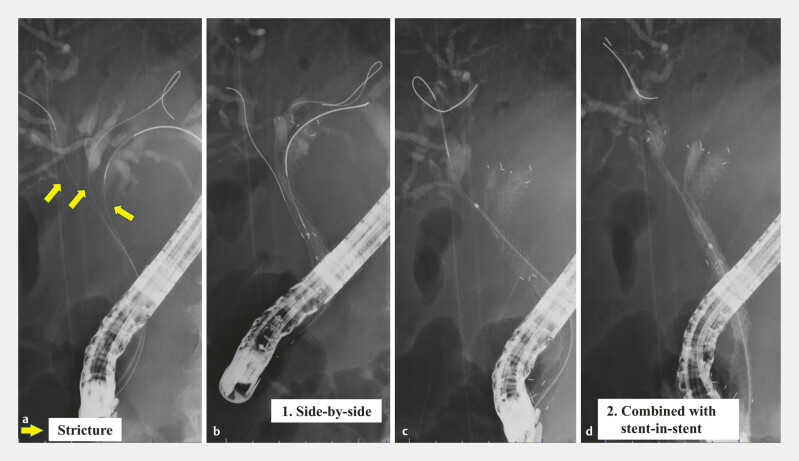
A procedure for stent placement. **a** Cholangiography revealed bismuth type IV, with multiple strictures observed in the right anterior hepatic duct. The yellow arrow indicates the site of stricture. **b** First, multi-hole self-expandable metal stents (MHSEMSs) are simultaneously deployed in the right and left bile ducts using the side-by-side technique. **c** Next, a guidewire is advanced into the target bile duct through a hole on the luminal side of the MHSEMS planned for the stent-in-stent technique. **d** Finally, after advancing the third MHSEMS into the target bile duct, it is deployed to complete the procedure.

A 72-year-old woman with hilar cholangiocarcinoma accompanied by peritoneal dissemination was undergoing chemotherapy. Plastic stents (PSs) had been placed in the right and left bile ducts ( Fig. 1 ); however, liver enzyme levels remained elevated. To allow the stable continuation of chemotherapy, the PSs were exchanged for SEMSs. Cholangiography revealed bismuth type IV, with multiple strictures observed in the right anterior hepatic duct. To achieve more effective biliary drainage, the placement of three SEMSs was planned. First, three guidewires (GWs) were advanced into the target bile ducts, and the MHSEMSs were simultaneously deployed in the right and left bile ducts using a side-by-side placement. Subsequently, a GW was advanced into the right anterior hepatic duct through a hole in the right MHSEMS, and a third MHSEMS was deployed using a stent-in-stent placement ( Fig. 2 ; Video 1 ). The procedure was completed without adverse events, and liver enzyme levels normalized.

Multi-hole two-way hybrid stent placement using three 8-mm metal stents for malignant hilar biliary obstruction.Video 1

**Fig. 3:**
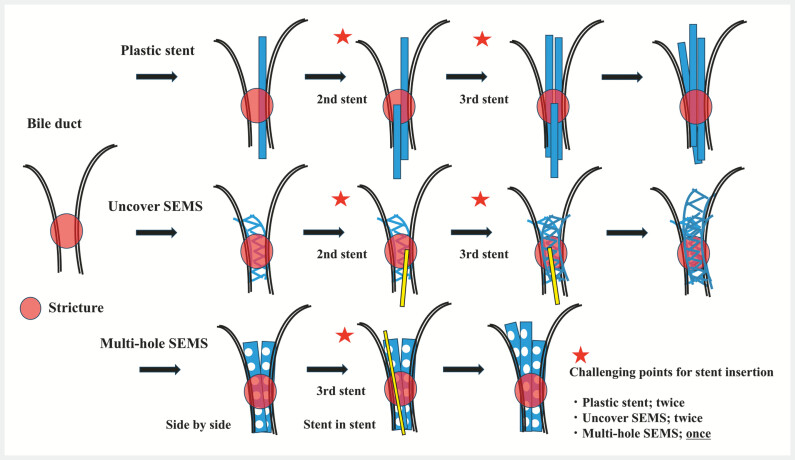
Technical challenges of trisegment drainage stent placement. The bile duct and tumor-related stricture are indicated by a red circle. Trisegment drainage generally requires the placement of three stents. There are technical challenges in placing three stents as planned, and this figure illustrates the issues associated with plastic stents (PSs), uncovered self-expandable metal stents (UCSEMSs), and multi-hole SEMSs (MHSEMS). With PSs, the insertion of the second and third stents can be technically difficult. With UCSEMSs, the insertion of the delivery system for the second and third stents becomes technically challenging because it must pass through the metallic mesh. In particular, delivery of the third stent is extremely difficult, as it needs to traverse multiple layers of metallic mesh. This difficulty is encountered with both the stent-in-stent technique and the side-by-side technique, since passage through multiple metallic meshes is required in either approach. However, with this technique using MHSEMSs, passage through the metallic mesh is required only once. In addition, the route for delivery of the third stent is limited, making the procedure easier than with other methods.

This method is expected to provide a longer patency period and a lower risk of tumor ingrowth 2 . Most importantly, because only a single passage through the stent mesh is required, this technique facilitates for trisegment drainage ( Fig. 3 ) and is removable 3 4 .

Endoscopy_UCTN_Code_TTT_1AR_2AZ
